# Supplementary material for: Baseline characteristics, management, and outcomes of 55,270 children and adolescents diagnosed with COVID-19 and 1,952,693 with influenza in France, Germany, Spain, South Korea and the United States: an international network cohort study
Source: medRxiv. 2020 Oct 30:2020.10.29.20222083. Preprint. [Version 1] doi: 10.1101/2020.10.29.20222083 (PMC7605587; doi:10.1101/2020.10.29.20222083)
Supplement: 1 [file NIHPP2020.10.29.20222083-supplement-1.pdf]

medRxiv preprint doi: https://doi.org/10.1101/2020.10.29.20222083; this version posted October 30, 2020. The copyright holder for this preprint (which was not certified by peer review) is the author/funder, who has granted medRxiv a license to display the preprint in perpetuity.
It is made available under a CC-BY 4.0 International license .

Supplementary Table 1. Overview of data sources contributing results.

Database Name	Country	Description	Contributed By
Columbia University Irving Medical Center (CUIMC)	United States	The clinical data warehouse of NewYork-Presbyterian Hospital/Columbia University Irving Medical Center, New York, NY, based on its current and previous electronic health record systems, with data spanning over 30 years and including over 6 million patients	Department of Biomedical Informatics, Columbia University Irving Medical Center, New York, NY 10032, USA
IQVIA Disease Analyser (DA) Germany	Germany	IQVIA DA Germany is collected from extracts of patient management software used by GPs and specialists practicing in ambulatory care settings. Data coverage includes more than 34M distinct person records out of at total population of 80M (42.5%) in the country and collected from 2,734 providers. Dates of service include from 1992 through March 2020.	Real World Solutions, IQVIA Inc, Cambridge, MA, USA
HealthVerity	United States	This HealthVerity derived data set contains de-identified patient information with an antibody and/or diagnostic test for COVID-19 linked to all available Medical Claims and Pharmacy Data from select private data providers participating in the HealthVerity marketplace.	Janssen Research & Development, Titusville, NJ, USA
Health Insurance Review & Assessment Service (HIRA)	South Korea	National claim data from a single insurance service from South Korea. It contains the observational medical records (including both inpatient and outpatient) of a patient while they are qualified to get the national medical insurance.	Health Insurance Review & Assessment Service, 60 Hyeoksin-Rho, Wonju-Si, Gangwon-Do(Bangkok-Dong), 26465, Korea

medRxiv preprint doi: https://doi.org/10.1101/2020.10.29.20222083; this version posted October 30, 2020. The copyright holder for this preprint (which was not certified by peer review) is the author/funder, who has granted medRxiv a license to display the preprint in perpetuity.
It is made available under a CC-BY 4.0 International license .

IQVIA Open Claims		A United States database of open, pre-adjudicated claims from January 2013 to May 2020. Data are reported at anonymized patient level collected from office-based physicians and specialists via office management software and clearinghouse switch sources for the purpose of reimbursement. A subset of medical claims data have adjudicated claims.	Real World Solutions, IQVIA Inc, Cambridge, MA, USA
IQVIA Longitudinal Patient Data (LPD) France	France	LPD France is a computerised network of physicians including GPs who contribute to a centralised database of anonymised patient EMR. Currently, >1200 GPs from 400 practices are contributing to the database covering 7.8M patients in France. The database covers a time period from 1994 through the present. Observation time is defined by the first and last consultation dates. Drug information is derived from GP prescriptions. Drugs obtained over the counter by the patient outside the prescription system are not reported.	Real World Solutions, IQVIA Inc, Cambridge, MA, USA
OPTUM-EHR	United States	Optum® de-identified COVID-19 Electronic Health Record dataset represents Optum's Electronic Health Record data a medical records database for patients receiving a COVID-19 diagnosis record or lab test for SARS-CoV-2. The medical record data includes clinical information, inclusive of prescriptions as prescribed and administered, lab results, vital signs, body measurements, diagnoses, procedures, and information derived from clinical Notes using Natural Language Processing (NLP).	Janssen Research & Development, Titusville, NJ, USA

medRxiv preprint doi: https://doi.org/10.1101/2020.10.29.20222083; this version posted October 30, 2020. The copyright holder for this preprint (which was not certified by peer review) is the author/funder, who has granted medRxiv a license to display the preprint in perpetuity.
It is made available under a CC-BY 4.0 International license .

Premier Healthcare Database	United States	The Premier Healthcare Database contains complete clinical coding, hospital cost, and patient billing data from approximately 700 hospitals throughout the United States representing 20% of inpatient hospital stays. Premier collects data from participating hospitals in its health care alliance. The Premier health care alliance was formed for hospitals to share knowledge, improve patient safety, and reduce risks. Participation in the Premier health care alliance is voluntary. Although the database excludes federally funded hospitals (e.g., Veterans Affairs), the hospitals included are nationally representative based on bed size, geographic region, location (urban/rural) and teaching hospital status. The database contains a date-stamped log of all billed items by cost-accounting department including medications; laboratory, diagnostic, and therapeutic services; and primary and secondary diagnoses for each patientâ€™s hospitalization.	Janssen Research & Development, Titusville, NJ, USA
Information System for Research in Primary Care (SIDIAP)	Spain	The Information System for Research in Primary Care (SIDIAP; www.sidiap.org) is a primary care records database that covers approximately 7 million people, equivalent to an 80% of the population of Catalonia, North-East Spain. Healthcare is universal and tax-payer funded in the region, and primary care physicians are gatekeepers for all care and responsible for repeat prescriptions.	Fundacio Institut Universitari per a la recerca a l'Atencio Primaria de Salut Jordi Gol i Gurina (IDIAPJGol), Barcelona, Spain

medRxiv preprint doi: https://doi.org/10.1101/2020.10.29.20222083; this version posted October 30, 2020. The copyright holder for this preprint (which was not certified by peer review) is the author/funder, who has granted medRxiv a license to display the preprint in perpetuity.
It is made available under a CC-BY 4.0 International license .

STAnford medicine Research data Repository (STARR-OMOP)	United States	STAnford medicine Research data Repository, a clinical data warehouse containing live Epic data from Stanford Health Care, the Stanford Children's Hospital, the University Healthcare Alliance and Packard Children's Health Alliance clinics and other auxiliary data from Hospital applications such as radiology PACS. STARR platform is developed and operated by Stanford Medicine Research IT team and is made possible by Stanford School of Medicine Research Office.	Department of Medicine, School of Medicine, Stanford University, Redwood City, CA USA
U of Colorado Anschuz Medical Campus Health Data Compass (CU-AMC-HDC)	United States	Health Data Compass (HDC) is a multi-institutional data warehouse. HDC contains inpatient and outpatient electronic medical data including patient, encounter, diagnosis, procedures, medications, laboratory results from two electronic medical record systems (UCHealth and Children's Hospital of Colorado), state-level all-payers claims data, and the Colorado death registry. Acknowledgement statement: Supported by the Health Data Compass Data Warehouse project (healthdatacompass.org).	Data Science to Patient Value Program, Department of Medicine, University of Colorado Anschutz Medical Campus, Aurora, Colorado, USA

medRxiv preprint doi: https://doi.org/10.1101/2020.10.29.20222083; this version posted October 30, 2020. The copyright holder for this preprint
(which was not certified by peer review) is the author/funder, who has granted medRxiv a license to display the preprint in perpetuity.
It is made available under a CC-BY 4.0 International license .

Supplementary Table 2. Cohort definitions and codes.

**Name**	**Atlas Link**
**COVID-19**	
Persons tested with a COVID-19 diagnosis record or a SARS-CoV-2 positive test with at least 365d prior observation	https://atlas.ohdsi.org/#/cohortdefinition/202
Persons hospitalized with a COVID-19 diagnosis record or a SARS-CoV-2 positive test with at least 365d prior observation	https://atlas.ohdsi.org/#/cohortdefinition/197
Persons tested with a COVID-19 diagnosis record or a SARS-CoV-2 positive test with no required prior observation	http://atlas-covid19.ohdsi.org/#/cohortdefinition/970
Persons hospitalized with a COVID-19 diagnosis record or a SARS-CoV-2 positive test with no required prior observation	http://atlas-covid19.ohdsi.org/#/cohortdefinition/974
**Influenza**	
Persons with Influenza diagnosis or positive test 2017-2018 with at least 365d prior observation	https://atlas.ohdsi.org/#/cohortdefinition/211
Persons hospitalized with influenza diagnosis or positive test 2017-2018 with at least 365d prior observation	https://atlas.ohdsi.org/#/cohortdefinition/212
Persons with Influenza diagnosis or positive test 2017-2018 with no required prior observation	http://atlas-covid19.ohdsi.org/#/cohortdefinition/958
Persons hospitalized with influenza diagnosis or positive test 2017-2018 with no required prior observation	http://atlas-covid19.ohdsi.org/#/cohortdefinition/961
**Comorbidities**	
Asthma	https://atlas.ohdsi.org/#/cohortdefinition/218
Heart disease	https://atlas.ohdsi.org/#/cohortdefinition/231
Hypertension	https://atlas.ohdsi.org/#/cohortdefinition/227
Malignant neoplasm excluding non-melanoma skin cancer	https://atlas.ohdsi.org/#/cohortdefinition/222
Obesity	https://atlas.ohdsi.org/#/cohortdefinition/224
Autistic disorder	https://atlas.ohdsi.org/#/concept/439780

medRxiv preprint doi: https://doi.org/10.1101/2020.10.29.20222083; this version posted October 30, 2020. The copyright holder for this preprint (which was not certified by peer review) is the author/funder, who has granted medRxiv a license to display the preprint in perpetuity.
It is made available under a CC-BY 4.0 International license .

Neonatal disorder	https://atlas.ohdsi.org/#/concept/4042220
Neurodevelopmental disorder	https://atlas.ohdsi.org/#/concept/45771096
Type 1 diabetes mellitus	https://atlas.ohdsi.org/#/concept/201254
Attention deficit hyperactivity disorder	https://atlas.ohdsi.org/#/concept/438409
Chromosomal disorder	https://atlas.ohdsi.org/#/concept/4257441
Congenital malformation	https://atlas.ohdsi.org/#/concept/4079975
Congenital heart disease	https://atlas.ohdsi.org/#/concept/312723
Prematurity of infant	https://atlas.ohdsi.org/#/concept/36675035
**Outcomes**	
**During hospitalization**	
Sepsis during hospitalization	https://atlas.ohdsi.org/#/cohortdefinition/277
Acute Respiratory Distress syndrome (ARDS) during hospitalization	https://atlas.ohdsi.org/#/cohortdefinition/278
Cardiac arrhythmia	https://atlas.ohdsi.org/#/cohortdefinition/248
Bleeding	https://atlas.ohdsi.org/#/cohortdefinition/238
**30-day outcomes**	
Death	http://atlas-covid19.ohdsi.org/#/cohortdefinition/166
Hospitalization episodes	http://atlas-covid19.ohdsi.org/#/cohortdefinition/917
Pneumonia	http://atlas-covid19.ohdsi.org/#/cohortdefinition/938
Multi-system inflammatory syndrome in children (MIS-C)	http://atlas-covid19.ohdsi.org/#/cohortdefinition/940

medRxiv preprint doi: https://doi.org/10.1101/2020.10.29.20222083; this version posted October 30, 2020. The copyright holder for this preprint (which was not certified by peer review) is the author/funder, who has granted medRxiv a license to display the preprint in perpetuity.
It is made available under a CC-BY 4.0 International license .

Supplementary Table 3. Demographics, comorbidities, symptoms and outcomes among diagnosed with seasonal influenza (2017-2018) among children/adolescents aged below 18 years*

	At least 1 year of prior observation					No prior observation time			
	SIDIAP (Spain)	IQVIA LPD (France)	CU-AMC HDC (US)	IQVIA OpenClaims (US)	OPTUM EHR (US)	CUIMC (US)	IQVIA DA (Germany)	HealthVerity (US)	STARR-OMOP (US)
	n=26929	n=22425	n=3339	n=1873480	n=3665	n=2112	n=19863	n=502	n=2831
**Age (years)**									
00-04	22.0	20.8	23.7	24.4	24.1	40.2	29.3	21.7	25.3
05-09	36.7	35.3	35.6	37.5	31.3	36.2	35.7	29.7	34.7
10-14	29.2	27.0	27.0	26.3	24.9	17.2	23.3	23.7	27.8
15-19	12.0	17.0	13.6	11.8	19.7	6.5	11.7	24.9	12.2
**Gender**									
Female	47.5	47.8	49.6	48.5	50.9	48.2	47.4	50.6	46.2
Male	52.5	51.9	50.4	51.5	49.1	51.8	52.6	49.4	53.8
**Comorbidities****									
Autistic disorder	0.5	-	0.9	1.0	1.4	-	-	-	-
Neonatal disorder	2.4	-	0.9	0.3	0.9	-	-	-	-
Neurodevelopmental disorder	6.7	0.6	3.5	6.4	10.2	-	-	-	-
Asthma	8.6	16.4	15.9	27.5	29.7	-	-	-	-
Obesity	8.3	1.5	3.4	4.2	13.9	-	-	-	-
Heart disease	1.8	0.4	2.3	4.5	7.2	-	-	-	-
Malignant neoplasm excluding non-melanoma skin cancer	0.1	0.1	-	0.5	3.5	-	-	-	-
Hypertension	0.1	0.1	0.4	1.1	1.2	-	-	-	-
Type 1 diabetes mellitus	0.1	0.0	-	0.2	0.4	-	-	-	-
Attention deficit hyperactivity disorder	2.4	0.0	1.7	4.5	6.7	-	-	-	-

medRxiv preprint doi: https://doi.org/10.1101/2020.10.29.20222083; this version posted October 30, 2020. The copyright holder for this preprint (which was not certified by peer review) is the author/funder, who has granted medRxiv a license to display the preprint in perpetuity.
It is made available under a CC-BY 4.0 International license .

Chromosomal disorder	0.1	-	0.4	0.3	0.8	-	-	-	-
Congenital malformation	11.2	0.4	2.5	2.7	4.7	-	-	-	-
Congenital heart disease	0.2	-	0.3	0.3	0.6	-	-	-	-
Prematurity of infant	0.6	-	0.4	0.2	0.6	-	-	-	-
**Symptoms at index date**									
Fever	1.7	15.9	42.1	27.9	38.6	51.8	9.3	31.3	40.6
Cough	0.2	10.4	21.9	11.4	14.4	16	4.1	16.9	11.8
Dyspnea	0.0	0.0	0.6	0.4	0.6	2.2	0.9	-	1.1
Malaise or fatigue	0.0	1.0	0.9	0.7	0.7	0.5	0.4	-	1.1
Myalgia	0.0	0.3	1.0	0.6	0.5	0.5	0.1	-	1.0
Anosmia OR Hyposmia OR Dysgeusia	-	-	-	0.0	-	-	-	-	-
Gastrointestinal tract symptoms	9.6	3.2	5.6	3.4	3.9	6.1	2.3	5.4	4.5
Diarrhea	0.6	0.0	1.3	0.5	0.8	0.9	-	-	0.8
Vomiting	1.1	2.0	3.9	2.8	2.9	4.9	1.9	4.4	3.4
Nausea	1.1	2.0	3.5	1.6	2.1	0.6	2.0	3.2	1.4
Bronchiolitis	0.2	0.2	1.0	0.5	0.4	0.8	0.0	-	1.1
**30-day outcomes during hospitalization**									
Sepsis	-	-	-	-	0.2	0.4	-	-	0.4
Acute respiratory distress syndrome (ARDS)	-	-	-	0.1	0.3	0.7	-	-	0.7
Cardiac arrhythmia	-	-	-	-	-	0.7	-	-	0.2
Bleeding	-	-	-	-	0.2	0.6	-	-	0.3
**30-day outcomes**									
Death	-	-	-	-	-	-	-	-	-
Hospitalization episodes	-	-	1.6	0.9	18.7	7.4	-	1.4	3.7
Pneumonia	0.7	0.4	2.2	2.4	1.4	3.1	2.0	-	2.4
Multi-system inflammatory syndrome in children (MIS-C)	-	0.0	-	0.0	-	-	0.0	-	-

medRxiv preprint doi: https://doi.org/10.1101/2020.10.29.20222083; this version posted October 30, 2020. The copyright holder for this preprint
(which was not certified by peer review) is the author/funder, who has granted medRxiv a license to display the preprint in perpetuity.
It is made available under a CC-BY 4.0 International license .

*Proportions presented among diagnosed or hospitalized patients by database (column percentage); - data not available or below the minimum cell count required (5 individuals); children aged <1 year were excluded when at least 1 year of prior observation time was required.

**Comorbidities are reported only in those databases with at least 1 year of prior observation time.

Abbreviations: Colorado University Anschuz Medical Campus Health Data Compass (CU-AMC HDC), Columbia University Irving Medical Center (CUIMC), Data Analyzer (DA), Health Insurance Review & Assessment Service (HIRA), Information System for Research in Primary Care (SIDIAP), STAnford medicine Research data Repository (STARR-OMOP), Longitudinal Patient Data (LPD).

Supplementary Table 4. Characteristics of diagnosed and hospitalized COVID-19 children/adolescents with no prior observation time in those databases with available observation time*

	Diagnosed					Hospitalized		
	SIDIAP (Spain)	IQVIA LPD (France)	CU-AMC HDC (US)	IQVIA OpenClaims (US)	OPTUM EHR (US)	HIRA (South Korea)	IQVIA OpenClaims (US)	OPTUM EHR (US)
	n = 5037	n = 979	n = 286	n = 17618	n = 7737	n = 251	n = 3055	n = 748
**Comorbidities**								
Autistic disorder	0.7	-	-	0.4	0.3	-	0.9	1.9
Neonatal disorder	1.7	-	-	1.9	0.8	-	7.8	5.7
Neurodevelopmental disorder	6.1	-	-	1.4	1.6	-	3.4	7.8
Asthma	-	-	-	0.9	0.7	-	1.4	2.8
Obesity	-	-	-	0.1	0.5	-	0.4	1.6
Heart disease	-	-	-	0.6	0.5	-	1.7	3.6
Malignant neoplasm excluding non-melanoma skin cancer	-	-	-	0.1	0.1	-	0.2	1.2
Hypertension	-	-	-	0.1	0.1	-	0.5	0.9
Type 1 diabetes mellitus	0.2	-	-	0.3	0.2	-	0.8	0.8
Attention deficit hyperactivity disorder	1.8	-	-	0.6	1.0	-	1.0	3.7
Chromosomal disorder	0.3	-	-	0.7	0.3	-	2.9	2.1
Congenital malformation	9.2	-	-	2.6	1.7	-	10.4	8.7

medRxiv preprint doi: https://doi.org/10.1101/2020.10.29.20222083; this version posted October 30, 2020. The copyright holder for this preprint (which was not certified by peer review) is the author/funder, who has granted medRxiv a license to display the preprint in perpetuity.
It is made available under a CC-BY 4.0 International license .

Congenital heart disease	0.2	-	-	0.6	0.3	-	2.8	1.9
Prematurity of infant	0.8	-	-	0.5	0.5	-	2.6	2.9
**Symptoms at index date**								
Fever	5.2	9.6	8.7	14.7	9.9	10	12.2	23.7
Cough	4.5	7.5	9.1	11.5	6.6	5.6	6.2	8.0
Dyspnea	0.3	-	4.5	4.6	1.7	9.6	11.2	7.0
Malaise or fatigue	-	1.6	-	1.1	1.0	-	1.0	1.3
Myalgia	-	-	-	0.3	0.6	-	0.2	1.2
Anosmia OR Hyposmia OR Dysgeusia	-	-	-	0.4	0.7	-	-	<0.7
Gastrointestinal tract symptoms	11.8	3.7	-	5.4	3.9	8.0	10.9	14.0
Diarrhea	3.7	-	-	1.6	1.7	-	2.4	5.1
Vomiting	1.7	1.6	-	3.2	1.9	2.4	7.5	7.8
Nausea	1.4	1.6	-	1.2	1.1	2.0	2.6	3.5
Bronchiolitis	1.3	-	-	6.6	1.1	-	21.9	8.0
**30-day outcomes**								
Death	-	-	-	-	-	-	-	-
Hospitalization episodes	0.5	-	3.8	12.6	6.4	100	97.7	100
Pneumonia	0.1	-	-	4.0	1.0	6.4	19.5	10.2
Multi-system inflammatory syndrome in children (MIS-C)	-	-	-	0.2	0.5	-	1.0	3.7
**30-day outcomes during hospitalization**								
Sepsis	-	-	-	1.2	0.7	-	6.0	7.9
Acute respiratory distress syndrome (ARDS)	-	-	-	3.1	1.0	-	16.1	10.7
Cardiac arrhythmia	-	-	-	0.9	0.3	-	5.0	2.9

medRxiv preprint doi: https://doi.org/10.1101/2020.10.29.20222083; this version posted October 30, 2020. The copyright holder for this preprint (which was not certified by peer review) is the author/funder, who has granted medRxiv a license to display the preprint in perpetuity.
It is made available under a CC-BY 4.0 International license .

Bleeding	-	-	-	0.3	0.3	-	1.9	3.3

*Proportions presented among diagnosed or hospitalized patients by database (column percentage); - data not available or below the minimum cell count required (5 individuals).

**Comorbidities are reported only in those databases with at least 1 year of prior observation time.

Abbreviations: Colorado University Anschuz Medical Campus Health Data Compass (CU-AMC HDC), Columbia University Irving Medical Center (CUIMC), Data Analyzer (DA), Health Insurance Review & Assessment Service (HIRA), Information System for Research in Primary Care (SIDIAP), STAnford medicine Research data Repository (STARR-OMOP), Longitudinal Patient Data (LPD).
